# The Efficiency of a Learning Software Compared to e-Books in Dental Education

**DOI:** 10.1055/s-0041-1735932

**Published:** 2021-12-14

**Authors:** Philipp Luhrenberg, Roman Kia Rahimi-Nedjat, Kawe Sagheb, Keyvan Sagheb, Bilal Al-Nawas

**Affiliations:** 1 Department of Oral and Maxillofacial Surgery, University Medical Center of the Johannes Gutenberg University, Mainz, Germany; 2 Department of Prosthodontics, University Medical Center of the Johannes Gutenberg University, Mainz, Germany

**Keywords:** dental education, e-learning, computer-based learning, learning style

## Abstract

**Objectives** Due to time-consuming curricular and extracurricular activities, students in dentistry and medicine can profit from efficient learning strategies. One strategy could be the preparation with individually designed educational software that embed different multimedia sources. The aim of this study was to determine the efficiency of such a program compared with an e-book similar to a traditional textbook.

**Materials and Methods** Dentistry students of the Johannes Gutenberg-University of Mainz passed an entrance multiple-choice test on the topic of odontogenic tumors and were then randomized into two groups. Afterward, both groups had 14 days to study on the topic of odontogenic tumors either with a learning software or an e-book. A final exam was then taken and the two groups were compared.

**Statistical Analysis** A least significant difference post hoc analysis comparing the group average values was performed. The level of significance was *p* <0.05.

**Results** Seventy-one students took part in the study. While students from the first and second clinical semester showed significantly better results and improvements with the e-book, an opposite effect was observed in students from the third and fifth clinical semester with significantly better results and improvements with the software.

**Conclusion** Depending on the clinical experience and knowledge, a multimedia educational software can help students in dentistry to enhance efficiency in the preparation for exams.

## Introduction

During the COVID-19 pandemic, dental curricula had to change their teaching method from face-to-face to online learning in a surreal time, the so-called “emergency remote teaching” 1 2 . Compared with traditional lecture, this “new” medium is not dependent on place, time, or topic which makes it much more flexible and scholar oriented, 3 4 5 and nowadays due to necessity to avoid dispensable contacts, e-learning is discussed as a healthy and safe alternative. 6 7 8

However, even though e-learning has been present for almost two decades and offers many possibilities, the integration in dental or medical education is dragging behind. 9 Taking into consideration, the short time to develop new modules, e-learning modules should be easy to create for trainers as well as efficient for trainees. Woelber et al demonstrated impressively that an easy-to-use software can overall lead to better test results as complex e-learning environments, which are more expensive and time-consuming during development. 10 In accordance with cognitive load theory, the question is how to only focus on the learners need and his or her preferred learning style. In 1987, Fleming et al developed the visual, auditory, reading/writing, and kinesthetic (VARK) model, a simplified learning theory which tries to declare how learner perceive and receive information from its learning environment: “visual,” “aural,” “read and write” and “kinesthetic” and their combination in a learners individuum. 11 Based on this understanding, every person has his or her own unique learning style (unimodal or multimodal), which in turn is one of the main challenges for a designed tool to interact and to deal with this diversity and to improve learning results. 12 Unfortunately, the distribution of VARK learning styles varies widely and depends on many variables such as gender, age, educational level, marital status, or sociocultural status varying in terms of number of modality (unimodal vs. multimodal) as well as the distribution of preferred learning styles themselves. 12 13 14 15 Subsequently, new learning tools should be investigated on students' academic performance periodically before implementing a new learning design in dental curriculum to guarantee its efficiency and motivation for dental students.

Hence, the aim of our study was to investigate whether an easy-and-quick-to-create software used for exam preparation could be more effective compared with a traditional e-book in general and to observe students in different academic years how to deal with different learning tools.

## Materials and Methods

Different methods were evaluated to create a software-like solution, which could be viewed on all devices (mobile or desktop and independent from the operating system) and contain embedded media. An intuitive User-Interface with clear arrangement and compact bundled information to make the word-count as low as possible were of paramount importance. For these reasons, we chose to create a multimedia PDF as this format offered all of our requirements. Furthermore, the topic odontogenic tumors was chosen as it is relevant for dental students and challenging to learn at the same time because of its infrequency and complexity. 16 17

**Fig. 1:**
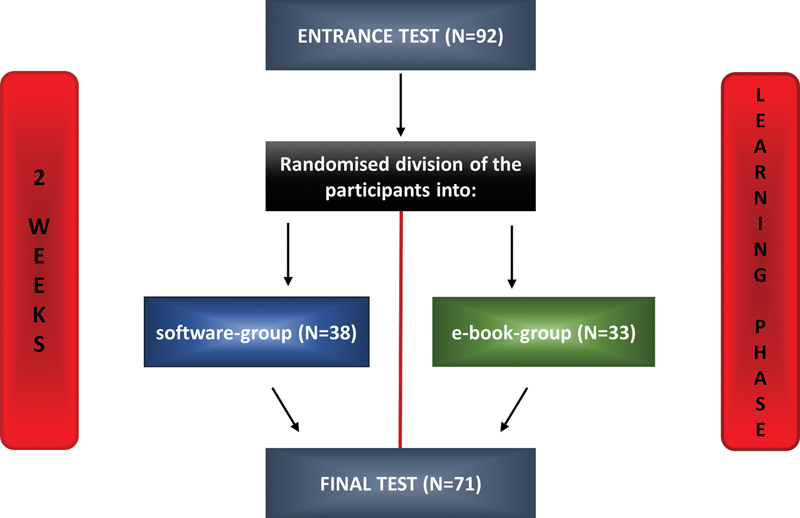
Study design.

A pretest and posttest procedure with one control group was performed ( Fig. 1 ). Students from four different semesters of the dentistry school of the Johannes Gutenberg-University of Mainz were included in the study (the first, second, third, and fifth semester of the clinical part of dental curriculum), and informed that participation was voluntarily and would not affect their course credits in any way.

After the pretest which consisted of 15 multiple choice questions about odontogenic tumors, all students were randomized into two groups. One group had access to download the software (e-learning tool [EL]) and the control group was able to download an e-book chapter about odontogenic tumors from the most frequently used textbook which was provided by the university library. It was important to offer the control group an e-book (EB) to eliminate the factors “time” and “place” which would be affected if the students had to carry around a traditional bound book. Both groups were given 14 days to spent time with the learning tool on their own, respectively and to prepare for the posttest which took place at the exact weekday and daytime (after 2 weeks). The posttest also consisted of 15 questions. Immediately after the posttest, a questionnaire was used to evaluate the students' learning styles, habits, and their opinion on the presented software.

In both tests, every correct answer was rated with one point so that the maximum number was 15 (only one answer was correct). False answers were not subtracted. Besides absolute numbers, we calculated the differences between the reached points from the posttest minus the reached points from the pretest to evaluate the absolute individual improvement. On one hand the results were calculated for all students according to the two learning groups. But on the other hand as the participants were from four different semesters reaching different “academic level,” the first two semesters were considered as those with less prior knowledge and the students from the last two semesters as those with better prior knowledge. These two groups were compared as well. Results were correlated by using the *t* -test or the Mann-Whitney U-test. A global significance level was chosen to be 0.05.

## Results

Seventy-one students took part in our study with a mean age of 25.1 years (±4.49 years; 35.2% males, 64.8% females) and completed pre- and posttest; initially, 91 participants passed the pretest (response rate 78.0%; for group assignment; Table 1 ). None of the students was able to reach the maximum number in each test (test 1: 1–12 and test 2: 3–14).

**Table 1:** Randomized group assignment

Academic level	group	EL	EB	Σ
1 + 2 clinical semester		23	17	40
3 + 5 clinical semester		15	16	31
Σ		38	33	71

Abbreviations: EB, e-book; EL, e-learning tool.

**Fig. 2:**
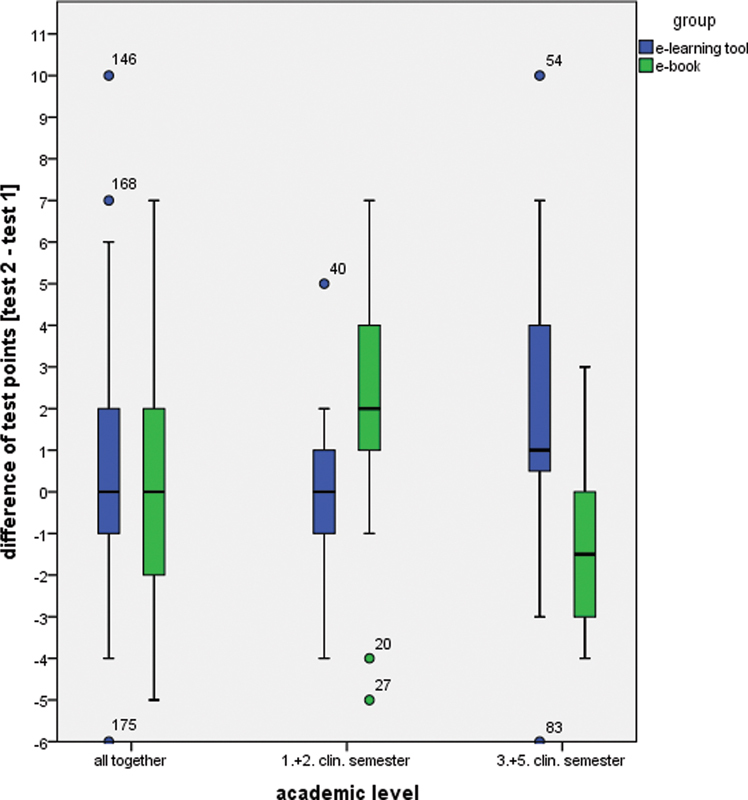
Boxplots showing the test difference between post- and pretest.

**Fig. 3:**
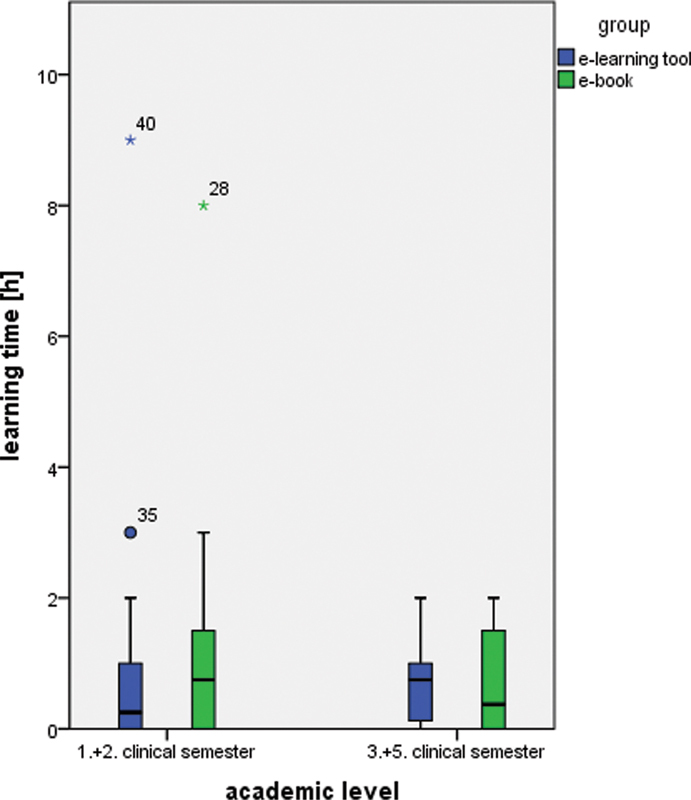
Boxplots showing the distribution of the learning time.

For all groups, the difference of the test results from the pre- to the posttest were moderately positive without a significant difference ( Fig. 2 ; Table 2 ). Moreover, the results were similar for the software group as well as for the e-book-group ( Fig. 2 ; Table 2 ). In tendency, test results were better for students who prepared with the software in the first and second clinical semester versus the participants from the third and fifth clinical semester. In fact, those tendencies for students learning with the software seem to be contrary to those learning with an e-book. To continue these inverse results amongst their academic level, students from the first and second clinical semester spent more time to prepare with the e-book in contrast to the remaining participants from the third and fifth clinical semester, considering the 15.5% of the entire cohort who did not answer this question ( Fig. 3 ; Table 3 ). Apart from their group assignment, students from the first and second clinical semester took more time (1.03 ± 1.98 hours) than their fellow students from the third and fifth clinical semester (0.70 ± 0.68 hours; *p* = 0.79).

**Table 2:** Test results as the difference between the points from the post- to the pretest

Academic level	group	*n*	Mean test points test 1 (points) (±SD)	Mean test points test 2 (points) (±SD)	*p* -Value a	Mean test difference (points) (±SD)	*p* -Value b
All together		71	7.46 (±2.40)	7.90 (±2.46)	0.24	0.44 (±3.11)	
	EL	38	7.13 (±2.17)	7.66 (±2.71)	0.303	−0.53 (±3.11)	0.968
	EB	33	7.85 (±2.62)	8.18 (±2.13)	0.547	−0.33 (±3.15)	
1 + 2 clinical semester	EL	23	6.96 (±2.06)	6.65 (±2.15)	0.009	−0.30 (±2.03)	0.015
	EB	17	6.24 (±2.39)	8.06 (±2.61)	0.518	1.82 (±3.23)	
3 + 5 clinical semester	EL	15	7.40 (±2.38)	9.20 (±2.83)	0.517	1.80 (±4.02)	0.013
	EB	16	9.56 (±1.59)	8.31 (±1.54)	0.985	−1.25 (±2.21)	

Abbreviations: EB, e-book; EL, e-learning tool; SD, standard deviation.

a *p* -Value for the difference between both tests.

b *p* -Value for the difference between both groups.

**Table 3:** Learning time (hours; students self-evaluation)

Academic level	group	*n*	Mean learning time (h) (±SD)	*p* -Value
1 + 2 clinical semester	EL	21	0.95 (±2.00)	0.378
	EB	17	1.29 (±1.99)	
3 + 5 clinical semester	EL	12	0.69 (±0.59)	0.974
	EB	10	0.73 (±0.80)	
All together		60	0.96 (±1.63)	

Abbreviations: EB, e-book; EL, e-learning tool; SD, standard deviation.

**Fig. 4:**
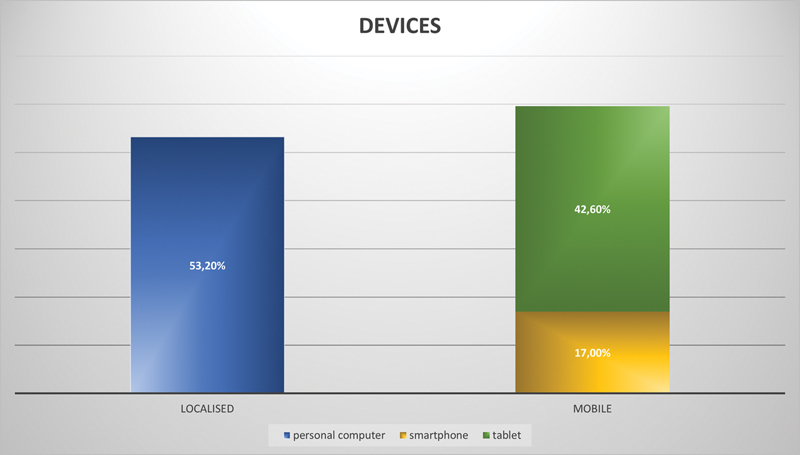
Distribution of media devices amongst dental students.

The questionnaires showed that over 70% of the participants used lectures or traditional utensils such as scripts and textbooks to prepare for an exam. Modern tools such as internet research (65.3%) or educational videos (22.5%) were used far less and mostly described as additional support. Their own home was the preferred learning environment for most students (78.9%), and only one quarter of the students stated that they like to learn in a group (26.8%). However, most students prepare their own learning material (93%), as these offer them adequate overview and preciseness. Apart from 2 weeks, students spent their time to prepare themselves immediately before the posttest, predominantly (67.7%). Both desktop computers and mobile devices were used equally often to perform internet research ( Fig. 4 ).

Self-evaluation according to the VARK model by Fleming et al showed that most participants had more than one learning style (71.8%, *n* = 71). Interestingly, those who prepared with the e-book most frequently chose “read and write” as their preferred style while students from the software group described themselves as multimodal learners. Overall, the students who had access to the software said that they would wish to use the software more frequently and for further topics.

## Discussion

Aim of our prospective, randomized clinical study was to evaluate the effect of a software on the learning efficiency in dental students from different semesters. The sample size in this study is comparable to similar studies or even higher. 18 19 20

In absolute numbers, students overall reached higher test points compared with the first test (positive results). Nevertheless, some students had lower test points in the posttest. The reason for that could be multifactorial. First, the learning topic for our trial were the odontogenic tumors who occur much less frequently than odontogenic cysts. 21 In accordance with cognitive load theory, the topic of the odontogenic tumors itself remains complex and takes a large amount from intrinsic cognitive load and is an unchangeable, but limiting factor for knowledge acquisition. 22 Second, it is possible that the questions in the second test were of higher difficulty (although the degree of the difficulty was assessed by two independent docents prior the study). Using a four-answer multiple choice test with one correct answer, there is a statistical probability approximately 25% to choose the right answer without prior knowledge. 23 Despite the likelihood for correct answering pre- and posttest without background knowledge, these data should be discussed comparing the groups among each other. 24

Interestingly, students with advanced knowledge show to some extent that they were able to improve significantly better by using the software than the e-book-chapter, while students with less prior knowledge performed significantly better by using the e-book. These findings are in accordance to most studies on computer-based learning as most study groups showed positive effects from the use of modern learning tools. 25 The influence of the individual knowledge is crucial to understand the benefit of these tools. Recent studies showed a significant difference in the results of participants from different educational levels. For example, Browne et al observed this effect between young dentists and well experienced post docs in a continuous professional development in health service 26 and Jager et al between medical students from different semesters when performing cases with virtual patients and their various diseases such as hepatitis or pneumothorax. 27 However, our study is the first to describe this finding for students from dentistry school.

Inverse effects like these could be explained with the adaption of learning styles, which can adapt due to educational time as the learning environment and lead to different student satisfaction. 28 29 Additionally, various learning tools and environments differently engage students satisfaction and influence individuals motivation to be willing to invest more learning time dealing with the offered learning content. 30 This explanation might be underlined by the outliners in Fig. 3 . Less time for preparation with the e-learning software could be due to the fact that one of our main demands for the software was to be precise in content with a low word count. While a running text from a book usually does not reveal all the information immediately, this is a true benefit of the software. Additionally, considering the fact that the reading speed on a screen is 25% less compared with a normal textbook puts the effectiveness of the software in terms of time saving in the right perspective. 23

Additionally, the learning program was commonly used on mobile devices as in general the use of mobile technologies seems to be a fundamental requirement to enhance and exploit all advantages of e-learning. 31 Although the participants specified that they had a positive effect from the use of the software most of the students indicated that visiting lectures would be the most preferred way to prepare for exams. However, with a lack of different opportunities lectures frankly still have to be the predominant didactic educational path 32 33 which is why e-learning unfortunately still plays a minor or subsidiary role in education 34 and students remain to be used to make paper-based notes of important information. This eventually explains the high portion of “read and write” as the preferred learning style in our questionnaire.

The fact that humans are used to adapt their learning style to their habits and offered opportunities can also be seen by the contrary answers from students, who had access to the software as they indicated to have different preferred learning styles more frequently. 33

Besides the above mentioned considerations, it is recommendable to further investigate learning environments. The limitation of our study are the small subgroups of different academic levels, lack of control to check the students information about their learning time (although some students frankly commented their low learning time), which could be eliminated—for example—to base on the data rate from the online platform the content is uploaded. 31 35 The strength of this study is the moment to place the study within the semester dissociated from exam phase and relevance to pass a test to guarantee the possibility to participate in a free and informal atmosphere.

This indeed shows that e-learning necessarily has to handle different learning styles and levels to be able to function as a unique tool for students from different semesters.

In conclusion, it can be said that the strength of e-learning tools is their versatility. With the inclusion of different media and contents, it is possible to generate a tool that can support students from many semesters and different learning styles.

Expressed with caution, it can be said, that learning with an e-book or a modified learning tool can lead to better test results and its use is a profitable medium. Before implementation of a new learning tool, its efficiency has to be proved to ensure individual learner's satisfaction and his or her learning outcome.
